# Supplementary material for: Synapse elimination activates a coordinated homeostatic presynaptic response in an autaptic circuit
Source: Commun Biol. 2020 May 22;3:260. doi: 10.1038/s42003-020-0963-8 (PMC7244710; doi:10.1038/s42003-020-0963-8)
Supplement: Supplementary file 1 — Supplementary Information [file 42003_2020_963_MOESM1_ESM.pdf]

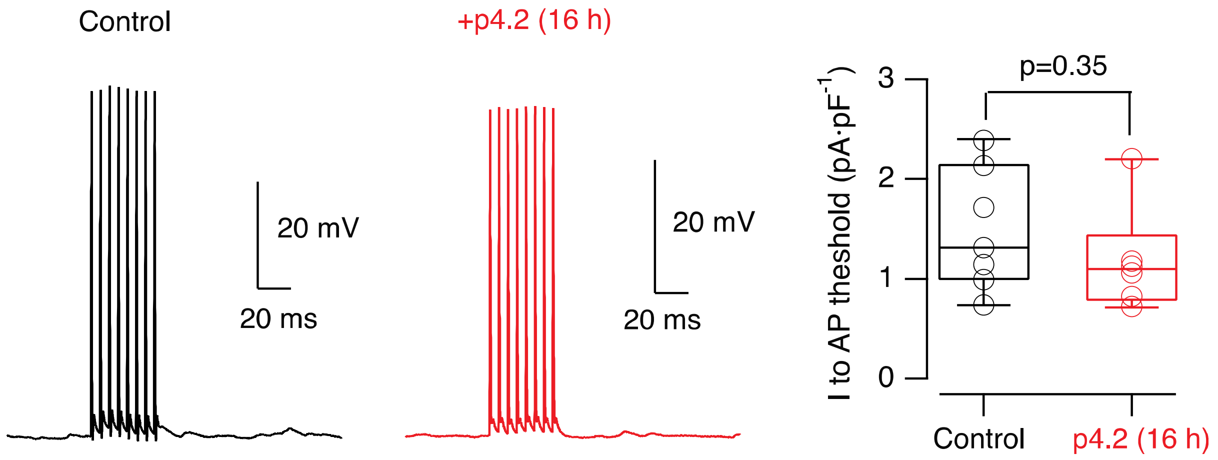


**Supplementary Figure 1. Exposure to p4.2 does not alter neuronal excitability.** Examples of action potentials evoked by current injections (8 stimuli delivered at 200 Hz) in a control neuron and in a neuron exposed to 200 nM p4.2 for 16 hours. The current required to trigger an action potential was comparable between untreated neurons (n=7) and neurons exposed to p4.2 for 16 h (n=6). Box plots show the median (horizontal line), 25 to 75% quartiles (boxes), and ranges (whiskers). Experimental groups were compared using the unpaired Student's t-test

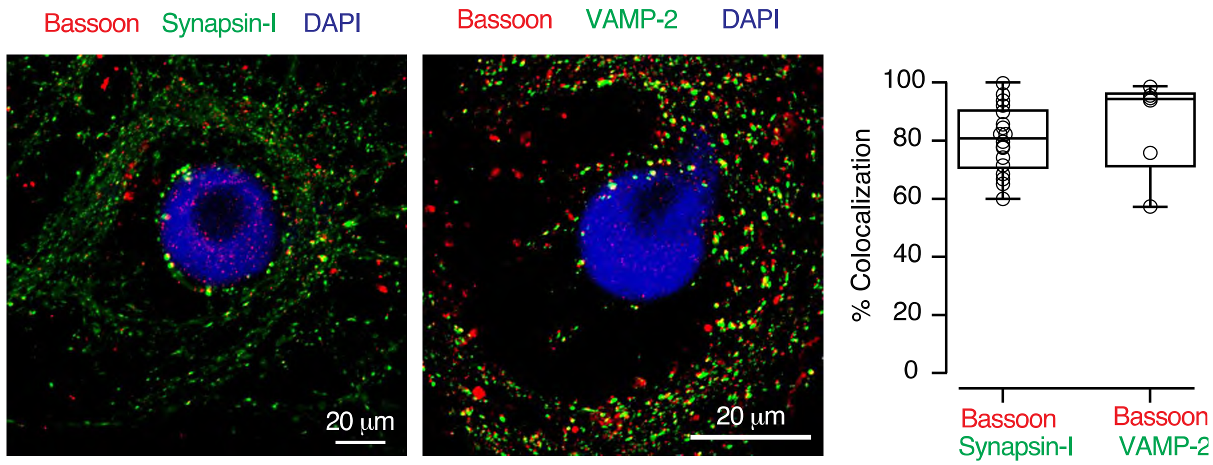


**Supplementary Figure 2. Co-localization of bassoon with synapsin-I and VAMP-2.** Example of two single cell microcultures (SCMs) stained for bassoon and synapsin-I or VAMP-2, respectively. 80±3% of bassoon puncta co-localized with synapsin-I (n=18) and 86±7% of bassoon puncta co-localized with VAMP-2 (n=6). Box plots show the median (horizontal line), 25 to 75% quartiles (boxes), and ranges (whiskers). The presence of a single neuron, as well as the absence of any other cell type in the microculture, is certified by DAPI staining.
