# Supplementary material for: Synapse elimination activates a coordinated homeostatic presynaptic response in an autaptic circuit
Source: Commun Biol. 2020 May 22;3:260. doi: 10.1038/s42003-020-0963-8 (PMC7244710; doi:10.1038/s42003-020-0963-8)
Supplement: Supplementary file 3 — Description of Additional Supplementary Files [file 42003_2020_963_MOESM3_ESM.pdf]

Description of Additional Supplementary Files

File Name: **Supplementary Movie 1**

Description: Changes in fluorescence visualized as ΔF/F0 in a neuron expressing SyGCaMP6f grown in a Single Cell Microculture (SCM). The movie displays a recording period of 3 s and each image is an average of 5 frames obtained at 40 Hz. Acquisition of images was combined with the simultaneous recording of excitatory postsynaptic currents (EPSCs) in the whole-cell configuration of the patch-clamp technique. The period of stimulation is indicated in the top left corner and consists in 5 stimuli delivered at 20 Hz.
